# Role of Host and Parasite MIF Cytokines during *Leishmania* Infection

**DOI:** 10.3390/tropicalmed5010046

**Published:** 2020-03-20

**Authors:** Thomas Holowka, Richard Bucala

**Affiliations:** 1 Department of Internal Medicine, Yale University School of Medicine, New Haven, CT 06520, USA; thomas.holowka@yale.edu; 2 Department of Pathology, Yale University School of Medicine, New Haven, CT 06520, USA; 3 Department of Epidemiology of Microbial Disease, Yale University School of Public Health, New Haven, CT 06520, USA

**Keywords:** *Leishmania*, MIF, inflammation, immunization

## Abstract

Macrophage migration inhibitory factor (MIF) is an immunoregulatory cytokine that has been extensively characterized in human disease and in mouse models. Its pro-inflammatory functions in mammals includes the retention of tissue macrophages and a unique ability to counteract the immunosuppressive activity of glucocorticoids. MIF also acts as a survival factor by preventing activation-induced apoptosis and by promoting sustained expression of inflammatory factors such as TNF-α and nitric oxide. The pro-inflammatory activity of MIF has been shown to be protective against *Leishmania major* infection in mouse models of cutaneous disease, however the precise role of this cytokine in human infections is less clear. Moreover, various species of *Leishmania* produce their own MIF orthologs, and there is evidence that these may drive an inflammatory environment that is detrimental to the host response. Herein the immune response to *Leishmania* in mouse models and humans will be reviewed, and the properties and activities of mammalian and *Leishmania* MIF will be integrated into the current understandings in this field. Furthermore, the prospect of targeting *Leishmania* MIF for therapeutic purposes will be discussed.

## 1. Brief Overview of Immune Response to *Leishmania*

The immune response during leishmaniasis has been actively studied for many years, and significant contributions to the field of immunology have been made in the study of the host response to *Leishmania* infection. A general understanding of immunity and leishmaniasis goes back even further, with the observation that some individuals who acquired cutaneous rashes at an early age were spared of more disfiguring or systemic disease later in life. The practice of leishmanization, a controlled intentional infection through exposure to sand flies or direct inoculation with infected tissue, has been practiced for centuries in endemic communities as a means of instilling long-lasting protective immunity [1]. Despite this well-established method of immunization, efforts at developing a formal human vaccine have fallen short, suggesting that gaps remain in the understanding of the parasite interaction with the host immune response.

Numerous studies in mouse models have elucidated the role of both the innate and adaptive immune response during *Leishmania* infection. Metacyclic phase promastigote stage parasites injected during the bite of an infected sand fly trigger an immediate host response. Neutrophils are initially recruited to the site of inoculation, where they phagocytose parasites but are ineffective at eliminating them, thus serving as a temporary cellular host [2,3]. It is believed that the parasites themselves are active in blocking host neutrophil apoptosis until infiltrating macrophages and dendritic cells arrive, and to phagocytose parasites and infected neutrophils alike [4,5,6]. These macrophages and dendritic cells go on to become the long-term hosts of amastigote stage parasites in cutaneous tissues [7,8].

The fate of amastigote stage parasites within the host phagocyte is dependent on the immune milieu and the consequential activation state of the host cell. Classically activated macrophages kill internalized parasites directly via production of nitric oxide (NO) and reactive oxygen species (ROS), or indirectly by undergoing apoptosis and thus eliminating the intracellular niche [7,9,10]. On the other hand, alternatively activated macrophages will not produce NO or ROS and instead upregulate arginase, promoting production of ornithine which may be scavenged by amastigotes and drive further growth [11,12]. Thus, blocking classical activation of host macrophages while promoting their long-term persistence are effective strategies to ensure parasite survival.

During infection with *Leishmania* a robust Th1-type adaptive immune response is necessary for activation of macrophages and destruction of internalized parasites. Infected and bystander dendritic cells are activated through TLR signaling to upregulate the co-stimulatory molecules CD40 and CD86 and produce the cytokine IL-12 [13,14]. The combination of co-stimulatory molecule binding and IL-12 signaling during antigen presentation to the immature CD4 T cell drives their differentiation into Th1-type CD4 T cells, which produce IFN-γ. IFN-γ signaling is crucial for classical activation of macrophages and dendritic cells, driving production of NO and elimination of internalized parasites [7,9]. This mechanism is well described in the C57BL/6 mouse model of infection. In contrast, in the BALB/c mouse model dendritic cells are incompletely activated and do not express IL-12, resulting in the establishment of a predominantly Th2-type T cell response [15,16]. These Th2 T cells produce IL-4 and IL-13 that promote alternative activation of macrophages, allowing for proliferation of internalized parasites [9,11]. Increased production of IL-10 by Th2 and Treg T cells also has been demonstrated to inhibit parasite destruction in mouse models of infection [17,18].

Lasting immunity to *Leishmania* is dependent on the generation of long-lived effector and memory CD4 and CD8 T cells [19,20]. Similar to other models of infection, during leishmaniasis most effector T cells succumb to apoptosis after the active phase of the immune response when the infection has been largely cleared, however a population of memory T cells persists [21,22,23]. These memory T cells are preselected through stimulation through the IL-7 receptor and will be reactivated to form effector populations during a subsequent infection [19,21]. It has additionally been proposed that following an infection with *Leishmania* the parasites are never completely eliminated and instead a persistent sub-clinical parasite burden exists that is necessary for host resistance to subsequent infection [9,18]. In the presence of this sub-clinical infection a population of long-lived effector CD4 T cells that express CD44 and IL7R exist [19,21]. In mouse models it has been demonstrated that both memory and long-lived effector T cell populations are critical for immunity to re-infection with *Leishmania*. 

Mouse models have provided crucial insights into the immune response to *Leishmania* infection, and similar mechanisms appear to apply to other infected mammalian hosts. Canines are considered to be a critical reservoir for *Leishmania*, and there is evidence that the majority of infected canines will progress to clinical disease. Similar to what has been observed in mice, a strong Th1 response marked by IFN-γ and TNF-α is associated with parasite control in clinically well canines, whereas those that develop a mixed Th1/Th2 response may progress to cutaneous and/or visceral disease [24]. Similarly, during cutaneous infection in humans there appears to be a mixed Th1/Th2 response, with limited evidence that IFN-γ is associated with increased healing [25]. However, in more severe forms of the disease including diffuse cutaneous, mucocutaneous, and visceral leishmaniasis, excessive production of inflammatory cytokines including IFN-γ, TNF-α and IL-17 may be associated with more severe disease [26,27,28,29]. These findings in humans and canines contrast with mouse models and suggest that a Th1-type response may not be entirely deleterious to parasite growth. Instead, a robust Th1 response may permit low level, subclinical infection in the case of cutaneous disease, or even drive an inflammatory environment associated with progressive disease and parasite proliferation in diffuse or visceral disease.

## 2. Function and Activity of Mammalian MIF

Among the many cytokines implicated in the immune response to *Leishmania*, the role of macrophage migration inhibitory factor (MIF) is particularly intriguing in that both the host and parasite produce their own orthologs during infection [30,31]. MIF is produced by a wide variety of organisms across the evolutionary spectrum, however virtually everything that is known about its activity is derived from studies of the mammalian protein.

**Figure 1:**
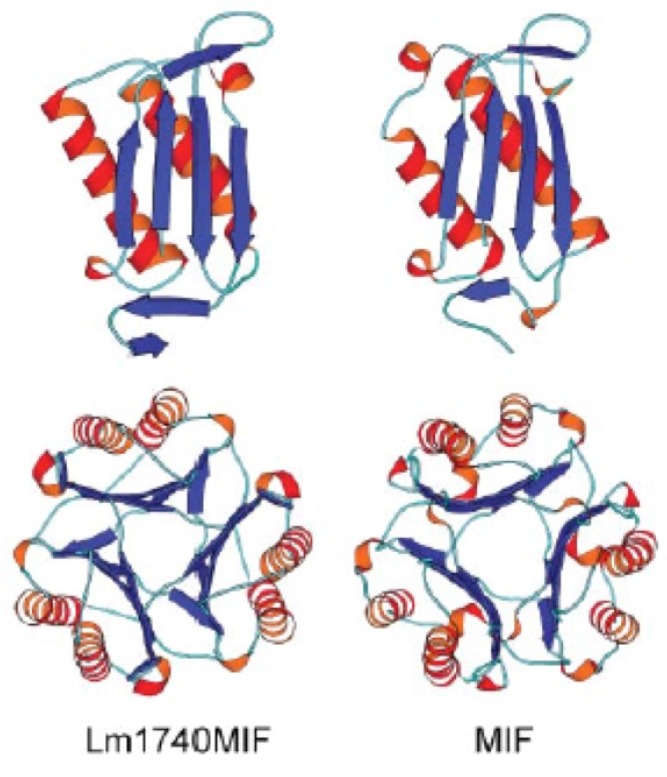
Schematic representation of *Leishmania*-encoded *Lm*1740MIF and human migration inhibitory factor (MIF) with monomer above and trimer below. β-strands in blue, α-helices in red, random coils in cyan. Taken from Kamir et al. 2008 [30].

The discovery of MIF dates back to the 1930s, when it was observed that immune cells did not egress from tuberculin-sensitized lymphoid tissue when placed in situ [32]. In 1966, this phenomenon was attributed to a factor elaborated by lymphocytes [33]. It was not until 1989 that a candidate gene was identified, and not until 1993 that it was cloned and expressed [34,35]. In 1996 the crystal structure of the molecule was solved, revealing a new cytokine superfamily comprising a barrel shaped trimer with each monomeric unit consisting of two α-helices above a sheet of four β-strands (Figure 1) [36,37]. 

In humans, MIF expression varies by a common polymorphism in the upstream promoter region of the gene. A variable number of CATT nucleotide repeats exist in this region, with 5–8 such sequences defining alternative alleles. The number of CATT repeats correlates with the constitutive and inducible expression of the mRNA and protein in the host [38]. MIF exists in preformed intracellular stores in various cell types, allowing for immediate release upon proper stimulation, with subsequent transcription triggered for ongoing secretion [39]. MIF signals primarily by binding to its cognate receptor, CD74, at the cell surface. This prompts the recruitment of surface CD44 and initiation of a signaling cascade that culminates in ERK1/2 phosphorylation [40,41]. MIF has further been shown to bind to intracellular Jab-1, promoting sustained ERK1/2 activity and enhanced PI3K/Akt signaling [42,43]. MIF has additionally been shown to bind directly to CXCR2 and CXCR4 and, by receptor de-sensitization, mediate migration arrest [44].

MIF was initially cloned from anterior pituitary cells and described as a pro-inflammatory counter-regulator of glucocorticoids [34]. It is now known to function as a broad upstream promoter of inflammation produced by a wide variety of mammalian cells, most notably immune effectors including T cells, macrophages, monocytes, dendritic cells, B cells and neutrophils [45]. MIF production is triggered by endotoxin stimulation and enhances expression of NO, prostoglandins, and various cytokines including TNF-α, IL-1β, IL-6, IL-8, and IL-12 [34,46,47]. MIF also has been shown to induce TLR4 expression as a means of further magnifying the endotoxin-triggered inflammatory response [48]. Other important activities downstream of MIF signaling include inhibition of glucocorticoid-mediated suppression of NFκB and MAPK, and suppression of p53 and Bad/FoxoA3-induced apoptosis of immune effector cells [43,49,50,51,52]. Lastly, MIF contributes to integrin activation and upregulation of ICAM-1 and VCAM-1 on monocytes [44,47,53]. Thus, the overall impact of MIF is to regulate immune effector migration to infected sites and promote their persistence while enhancing expression of pro-inflammatory molecules and cytokines.

The pro-inflammatory impact of MIF has been confirmed in studies showing the detrimental role of the cytokine in various inflammatory and autoimmune conditions. In humans, increased expression of MIF has been linked to pathogenesis in inflammatory conditions across organ systems, including atherosclerosis, asthma, cystic fibrosis, IBD, nephrotic syndrome, multiple sclerosis, rheumatoid arthritis, and lupus [54,55,56,57]. In patients suffering from sepsis MIF was found to be upregulated 5–10 fold, and higher MIF levels were found in individuals who ultimately died in comparison to those who lived [58,59]. In various studies, increased MIF expression has been found to correlate with increased production of inflammatory cytokines including TNF-α, IL-1, and IL-8 [54,60,61]. These observations are further supported by studies in MIF-knockout (MIF-KO) mice, showing reduced cytokine production and disease pathogenesis in the absence of MIF in different models of inflammatory and autoimmune disease [45]. Finally, MIF has been proposed as a tumorigenic factor, with upregulation seen in various cancers and increased MIF expression found to correlate with worse prognosis [62,63,64]. While much of the tumorigenic activity of MIF is likely related to its general pro-inflammatory effects, it also can function to directly promote tumor cell survival through inhibition of p53 and various other tumor suppressor genes in breast, bone, colorectal, and pancreatic cancer [65,66].

The prevalence of high expression *MIF* alleles in different human populations is indicative of the important protective effect this cytokine plays in infections where loss of pathogen control produces lethality [54]. Low expression of MIF was found to correlate with increased severity of *Mycobacterium tuberculosis* infection, and this finding was supported by studies of MIF-KO mice, which were found to be more susceptible to mycobacterium infection and deficient in their ability to eliminate internalized parasites [67,68]. Studies in MIF-KO mice have additionally shown increased susceptibility to infection with *Salmonella typhimurium*, *Toxoplasma gondii*, and *Trypanasoma cruzi* in the absence of MIF [69,70,71,72]. These studies suggest a role for MIF in controlling intracellular bacteria and parasites. In contrast, MIF expression may be detrimental during infection with certain viruses and extracellular bacteria. MIF deficiency was found to be protective in mice infected with West Nile virus, dengue virus, *Pseudomonas aeruginosa*, and *Helicobacter pylori* [73,74,75,76]. Similarly elevated MIF levels correlated with worse disease in humans infected with HBV, West Nile virus, dengue virus, *Streptococcus pneumonia*, and *H. pylori* [73,77,78,79,80]. 

In the case of malaria, MIF-KO mice were found to be protected from infection with *Plasmodium chabaudi*, with improved Th1-type response and reduced anemia [81,82,83]. Increased MIF expression correlated with death in patients with cerebral malaria as well as the development of placental malaria which is in accord with the role of MIF in the inflammatory clinical sequelae of *Plasmodium* infection [84,85]. Additionally, studies showed high expression *MIF* alleles to be associated with increased severity of anemia during malaria [86,87,88,89]. In light of these studies it has been proposed that malaria infection has driven the preponderance of low-expression *MIF* alleles in sub-Saharan African populations in comparison with Caucasians [54].

## 3. Role of Host MIF during *Leishmaniasis*

The role of MIF as a pro-inflammatory cytokine promoting TNF-α, NO and Th1-type immunity and mediating protection from intracellular pathogens has been well established. In this light it would be predicted that host MIF would have an anti-parasitic effect during *Leishmania* infection. Indeed it was shown in in vitro studies that addition of MIF to murine macrophages infected with *L. major* in culture resulted in increased parasite killing in a TNF-α and NO dependent manner [90]. A subsequent study showed that MIF promoted HIF-1α and NADPH-oxidase dependent killing of *L. amazonensis* by murine macrophages cultured in hypoxic conditions [91].

These in vitro studies suggest a role for MIF in promoting *Leishmania* killing by mouse macrophages. In support of this model, it was observed that MIF-KO mice on the usually resistant C57BL/6 background experienced worsened cutaneous disease and parasite burden than wild type mice. In this study, it also was observed that macrophages from MIF-KO mice were impaired in their ability to produce NO and ROS in response to IFN-γ, and consequently showed reduced parasite killing in vitro. Of note, it further was observed that MIF did not effect the development of a Th1 versus Th2 response and instead its protective effects were mediated entirely through macrophage activation [92]. Subsequent studies found that forced MIF expression by a *Salmonella* vector had a protective effect during experimental *L. pifanoi* infection [93]. Additionally, MIF production by CD4 T cells was found to be critical to protection in an experimental vaccination with *L. pifanoi* P4 antigen [94]. It should be noted that there were early reports that MIF could activate human and mouse macrophages to kill *L. donovani* in vitro which were subsequently retracted [95,96].

While the impact of host MIF on experimental *L. major* infection in mice has been well characterized, the impact of the cytokine in human leishmaniasis is less well understood. In one study, it was reported that the -173C single-nucleotide promoter polymorphism that is in linkage disequilibrium with the high expression *MIF* CATT-7 allele was associated with increased cutaneous disease severity in patients from Brazil infected with *L. braziliensis*, while in a separate study no difference in visceral leishmaniasis disease severity was observed in Indian individuals infected with *L. donovani* with differing *MIF* gene variants [97,98]. Another study found that plasma MIF and lipopolysaccharide levels were elevated in patients with American visceral leishmaniasis co-infected with HIV, and corresponded with a weakened T cell response and worsened systemic inflammation [99]. Thus, in contrast to the protective role of host MIF demonstrated in mice, MIF production in humans may correlate with worsened inflammation and disease during cutaneous and visceral leishmaniasis.

## 4. Overview of MIF Orthologs in *Leishmania* and Other Parasites

Understanding of the role of host MIF during leishmaniasis is confounded by the existence of parasite-encoded MIF orthologs that are produced by many species of *Leishmania* during infection. The discovery of *Leishmania*-encoded MIF orthologs is not entirely surprising, as MIF orthologs are found in organisms at all positions of the evolutionary tree, including protozoa, nematodes, plants, and invertebrate and vertebrate mammals alike. Furthermore, MIF orthologs have been reported in dozens of species of protozoan and helminthic parasites, and their properties have been thoroughly explored [100,101,102].

**Figure 2:**
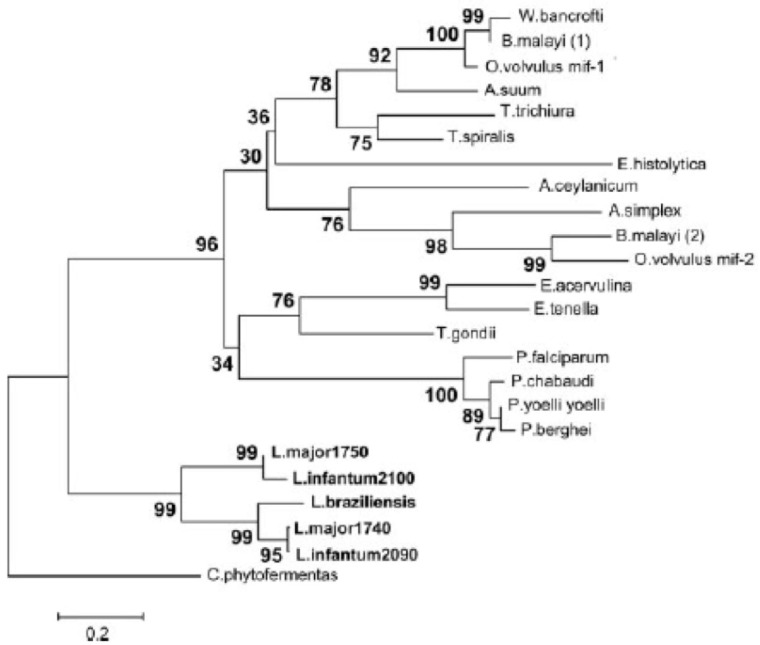
Phylogram of parasitic MIF protein sequences including five MIF-like proteins identified in the *Leishmania* species *L. major*, *L. infantum*, and *L. braziliensis*. The percentage of replicate trees in which the associated taxa clustered together in a bootstrap test are represented next to the branches. The evolutionary distances represented in the units of the number of amino acid substitutions per site as computed using the Poisson correction method. Taken from Kamir et al. 2008 [30].

Among kinetoplastid parasites that cause human disease, various species of *Leishmania* have been found to harbor MIF orthologs, while *Trypanasoma brucei and Trypanasoma cruzi* do not. Two MIF orthologs each are found in the genomes of species of the sub-genus *L. (Leishmania)*, including *L. major*, *L. mexicana*, and *L. infantum* (Figure 2). In contrast, no members of the *L. (Viannia)* sub-genus have been found to harbor MIF orthologs, and the *L. braziliensis* genome has two remnants of MIF genes, one with a deleted start codon and the other a truncated pseudogene [30,31]. These findings are suggestive of a selective pressure against MIF production in certain species of *Leishmania*, and furthermore that the gene is unnecessary to the survival of these species.

Among the *Leishmania* MIF orthologs, the two genes encoded by *L. major* are the most thoroughly studied. *Lm*1740MIF and *Lm*1750MIF are only 424 bp apart on chromosome 33 and encode proteins of 112 amino acids each (12.5 kD). They share 58% sequence identity with each other and 22% identity with human MIF [30,31]. Expression of mRNA for both genes is highest in procyclic promastigote stage parasites, with a 10-fold lower expression in the metacyclic phase and 2–5 fold lower expression in the amastigote stage [30]. In contrast, protein levels of both *Lm*1740MIF and *Lm*1750MIF are highest in amastigote stage parasites, a discrepancy that may be attributable to the high level of post-transcriptional regulation of *Leishmania* protein production [31,103].

Crystal structures have been solved for both *Lm*1740MIF and *Lm*1750MIF, showing close similarity to the human MIF protein structure (Figure 1) [30,31]. Purified recombinant native *Lm*1740MIF has tautomerase activity for the model substrate D-dopachrome methyl ester that is approximately 13-fold lower than human MIF. Furthermore, it binds human CD74 with a high affinity with a K_d_ of 2.9 × 10^−8^ M that is ~3-fold lower than human MIF (K_d_ = 9 × 10^−9^ M). In vitro, *Lm*1740MIF replicates many of the activities of mammalian MIF. It can regulate human PBMC chemotaxis, and it has been found to induce ERK1/2 phosphorylation in mouse macrophages while blocking p53 phosphorylation and NO-induced apoptosis. Furthermore, *Lm*1740MIF-induced ERK1/2 activation and p53 inhibition was abrogated in CD74-deficient macrophages [30]. These findings suggest that *Leishmania* MIF orthologs share many of the properties and activities of mammalian MIF in vitro, a paradoxical finding given the anti-parasitic role of host MIF proposed in mouse models of infection. 

The role of *Leishmania* MIF orthologs was further elucidated in studies using a strain of *L. major* in which both MIF genes had been deleted (*mif-/- L. major*). These parasites grew just as well as wildtype in the promastigote stage in vitro and were just as effective at infecting and persisting as amastigotes in mouse macrophages. Thus *Leishmania* MIF proteins are unlikely to play an essential role in developmental and survival of parasites. However, *mif-/- L. major* were more rapidly cleared by macrophages activated with LPS in vitro, and this was found to be dependent on more rapid apoptosis of macrophages infected in the absence of *Leishmania* MIF. It was therefore proposed that *Leishmania* MIF prevents host cell apoptosis and clearance of internalized parasites, and it was additionally found that this activity is dependent on host CD74 expression but independent of host NO production [104].

The ability of *Leishmania* MIF to regulate host macrophage activity was further demonstrated by microarray gene expression analysis and by flow cytometry showing differential expression of a number of immune factors in host cells infected in the presence or absence of the parasite cytokine. Among these, MHC II, CD86 and TNF-α were more highly expressed in the presence of *Leishmania* MIF, and it was furthermore found that T cell priming and activation was increased in dendritic cells infected with wildtype versus *mif-/- L. major.* Thus, *Leishmania* MIF has the paradoxical effect of augmenting T cell activation in vitro, an effect that was reflected in vivo in the increased expansion and activity of effector CD4 T cells in mice infected with wild type in comparison to *mif-/- L. major.* There was, however, a corresponding acceleration of CD4 T cell exhaustion in the presence of *Leishmania* MIF as indicated by a greater proportion of PD-1 positive CD4 T cells and reduced expression of IL-7, IL-7R and IFN-γ expression late in infection. This corresponded with increased parasite burden in mice infected with wildtype versus *mif-/- L. major* late in infection, suggesting a reduction in the formation of long-lived effector CD4 T cells in the presence of *Leishmania* MIF [104].

**Figure 3:**
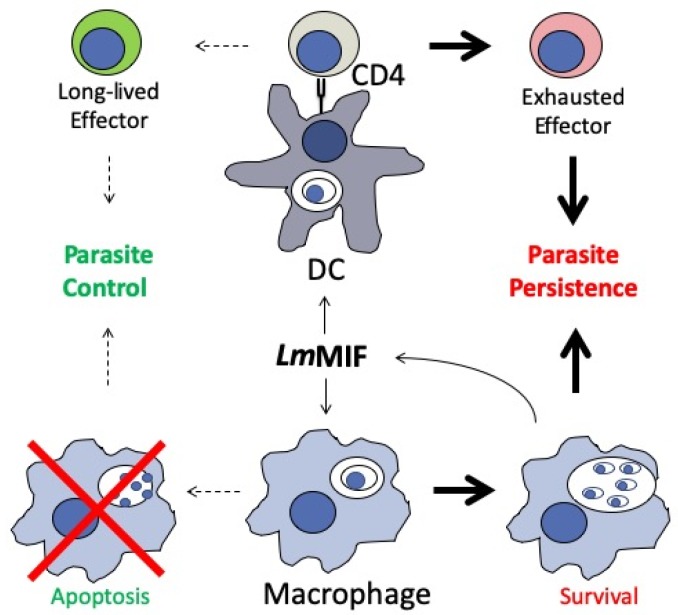
*Leishmania* MIF signals through the CD74 receptor on host macrophages and dendritic cells. It blocks macrophage apoptosis and simultaneous promotes dendritic cell-mediated T cell activation, resulting in increased T effector cell exhaustion and ultimately prolonged parasite persistence. Adapted from Holowka et al. 2016 [104].

At this time studies of *Leishmania* MIF indicate a complex role for the cytokine in simultaneously protecting the host cell niche while driving a hyper-inflammatory state in which effector T cells succumb to early exhaustion and apoptosis (Figure 3). The latter impact of *Leishmania* MIF is to promote long-term persistence of parasites and impact the ability of the host to form an effective memory response and avoid re-infection. Indeed, a similar role for parasite MIF in inhibiting T cell memory responses and preventing long-term immunity has been found in *Plasmodium* infection. In mouse models of infection with *Plasmodium berghei*, mice infected with MIF-deficient parasites were able to form increased numbers of memory CD4 T cells, had improved B cell and antibody responses, and were protected from future re-infection, whereas mice infected with wildtype parasites did not mount a memory T cell response and were susceptible to reinfection [105]. From these studies there emerges a paradigm in which parasite MIFs augment the host immune response in a manner that preserves the parasite niche and prevents development of long-term immunity.

The properties of over 20 MIF orthologs encoded by protozoan and helminthic parasites have been explored and reported on, and like *L. major*-encoded MIF these closely replicate many of the activities of mammalian MIF [101,102,106,107,108]. While *Leishmania* and *Plasmodium*-encoded MIF orthologs promote an inflammatory state, Th1-type response, orthologs of various helminthic pathogens drive an immunosuppressive, Th2-type response. In particular *Brugia malayi*-encoded MIF orthologs were found to enhance IL-4 induced alternative activation of macrophages, while *Anisakis simplex* encoded MIF promoted T regulatory cell recruitment and IL-10 production in vivo [109,110,111,112,113]. With these studies in mind, the impact of parasite encoded MIFs appear to be dependent on the immunologic context of the infection, with the ultimate effect of augmenting an immune response that is beneficial to parasite survival. With this in mind it is important to recognize that all of the studies of *Leishmania* MIF to this point have concerned *L. major* MIF in the context of a mouse model of cutaneous infection. It could be predicted that the pro-inflammatory impact of *Leishmania* MIF cytokines may be beneficial in promoting parasite persistence of cutaneous disease-causing species whereas the role for the cytokine may be different in species causing mucocutaneous and visceral disease. Indeed, the lack of MIF genes in species of the *L.* (*Viannia)* subgenus suggests a potentially detrimental role of the cytokine to survival of these organisms.

## 5. Targeting MIF for Therapeutic Applications

While further studies are needed to elucidate the mechanism by which *Leishmania* MIF modifies the host immune response, it is reasonable to consider that inhibition of the parasite cytokine could result in significant therapeutic benefit. Pharmacologic inhibitors of host MIF and CD74 have been studied extensively and have already advanced into human trials for treatment of SLE, multiple sclerosis and multiple myeloma [54,114,115]. Most small molecule inhibitors have been developed to target the tautomerase active domain that overlaps structurally with the CD74 binding region of the cytokine [116,117]. Critical differences in residues around these sites have been found in the crystal structures of various parasite MIF orthologs, including the structure of *L. major*, resulting in reduced or absent inhibitory effect [30,118,119,120]. These findings suggest structural dissimilarities that may allow for development of inhibitors with specificity for parasite rather than human MIF. 

Parasite MIF specific small molecule inhibitors have been reported in several studies. High throughput screening was used to identify a small molecule that selectively targeted *A. ceylanicum* MIF, inhibiting CD74 binding and chemotactic activity of the molecule and even killing *A. ceylanicum* in vitro without impacting human MIF function. A virtual screen was used to identify selective *P. falciparum* MIF inhibitors that prevented binding to CD74 without impacting human MIF activity [121,122]. These studies demonstrate the feasibility of producing parasite MIF specific inhibitors that do not impact host MIF activity, an important prospect in consideration of a disease such as leishmaniasis in which the parasite and human cytokines may have differing impacts on disease progression.

A separate approach to specific inhibition of parasite rather than host MIF is through immunization. This approach was utilized in a model of hookworm infection in which hamsters immunized with *A. ceylanicum* MIF had reduced weight loss and anemia in comparison to controls [123]. A similar approach was used in studies in which vaccination with a DNA plasmid encoding *Trichinella spiralis* MIF or with a *T. spiralis* MIF fusion protein promoted Th1 responses and partial protection in infected mice [124,125]. Similarly, vaccination with *Toxoplasma gondii* MIF was shown to promote antibody and T cell responses, improve survival times and reduce brain cyst formation in infected mice [126]. The most impressive impact of vaccination against parasite MIF was shown in a study in which mice were immunized with a *Plasmodium berghei* MIF-encoding RNA vector prior to infection with blood or liver stage parasites. In comparison to control immunization, these mice developed robust CD4 and CD8 memory T cell, T follicular helper cell, plasma cell, and anti-plasmodium antibody responses, were partially protected from initial infection, and were completely protected from a subsequent re-infection [127]. 

The success of pharmacologic inhibition of human MIF in clinical trials and parasite MIFs in vitro and in animal models demonstrates the potential for *Leishmania* MIF as a viable drug target. Current therapies for leishmaniasis such as pentavalent antimonials and liposomal amphotericin B are difficult to administer, have high side effect burdens, and carry increasing concern for resistance [128]. Thus, development and testing of *Leishmania* MIF inhibitors may prove an effective strategy for targeted treatment of leishmaniasis. Additionally, the prospect of targeting *Leishmania* MIF for vaccination is promising. Immunization with parasite antigens has proved successful in commercially available vaccines for canines including Leishmune® and Canileish®, however similar approaches have been less promising in humans, and thus a more sophisticated strategy to promote immunity may be necessary [129]. Because *Leishmania* MIF appears to play a role in reducing long-term immunity, immunization with the cytokine could prove a successful strategy for inducing protection to initial and recurrent infection. Vaccination studies in animal models should be pursued to determine the potential for use of *Leishmania* vaccines in humans.

## 6. Summary

In mouse models of leishmaniasis a protective immune response depends on a finely tuned Th1-type T cell response in order to direct macrophage killing of internalized parasites, and excessive inflammation can lead to T cell exhaustion and death, preventing sustained immunity. Similarly, in humans a Th1-type response appears to be protective in cutaneous disease, however excessive inflammation and T cell activity is associated with worsened visceral and diffuse disease., MIF is an inflammatory cytokine produced by mammalian immune effector cells that can promote CD4 T cell and macrophage activity, and has thus been shown to enhance clearance of *L. major* parasites in vitro and in vivo, and ameliorate disease in a mouse model of cutaneous leishmaniasis. However, in humans MIF has not been shown to have a protective effect during leishmaniasis and may in fact be detrimental. Moreover, many species of *Leishmania* produce orthologous versions of MIF, which have very similar structure and function to human MIF. These MIF proteins prolong survival of macrophage host cells and additionally promote parasite survival in mouse models by enhancing exhaustion and death of CD4 T cells. Similar activities have been shown in the case of other parasite MIF cytokines, particularly *Plasmodium* MIF, and vaccination against this cytokine has shown dramatic effects in improving host immunity and parasite clearance. With this in mind *Leishmania* MIF should be viewed as a promising target for immunization and pharmacologic inhibition in an effort to develop effective strategies for combating leishmaniasis.
